# Relevance of biomarkers across different neurodegenerative 

**DOI:** 10.1186/s13195-020-00601-w

**Published:** 2020-05-13

**Authors:** Alexander J. Ehrenberg, Ayesha Khatun, Emma Coomans, Matthew J. Betts, Federica Capraro, Elisabeth H. Thijssen, Konstantin Senkevich, Tehmina Bharucha, Mehrsa Jafarpour, Peter N. E. Young, William Jagust, Stephen F. Carter, Tammaryn Lashley, Lea T. Grinberg, Joana B. Pereira, Niklas Mattsson-Carlgren, Nicholas J. Ashton, Jörg Hanrieder, Henrik Zetterberg, Michael Schöll, Ross W. Paterson

**Affiliations:** 1 Memory and Aging Center, Weill Institute for Neurosciences, University of California, San Francisco, San Francisco, CA USA; 2 Department of Integrative Biology, University of California, Berkeley, Berkeley, CA USA; 3 Helen Wills Neuroscience Institute, University of California, Berkeley, Berkeley, CA USA; 4 Dementia Research Centre, University College London Institute of Neurology, London, UK; 5 Department of Radiology & Nuclear Medicine, Amsterdam UMC, location VUmc, Amsterdam Neuroscience, Amsterdam, The Netherlands; 6 German Center for Neurodegenerative Diseases (DZNE), Magdeburg, Germany; 7 Institute of Cognitive Neurology and Dementia Research, Otto von Guericke University Magdeburg, Magdeburg, Germany; 8 The Francis Crick Institute, London, UK; 9 Department of Neuromuscular Diseases, University College London Queen Square Institute of Neurology, London, UK; 10 Department of Clinical Chemistry, Amsterdam UMC, Amsterdam, The Netherlands; 11 Petersburg Nuclear Physics Institute names by B.P. Konstantinov of National Research Center, Kurchatov Institute, St. Petersburg, Russia; 12 First Pavlov State Medical University of St. Petersburg, St. Petersburg, Russia; 13 Oxford Glycobiology Institute, Department of Biochemistry , University of Oxford, Oxford, UK; 14 Department of Neurodegenerative Disease, UCL Queen Square, Institute of Neurology, University College London, London, UK; 15 Department of Psychiatry and Neurochemistry, Sahlgrenska Academy at the University of Gothenburg, Mölndal, Sweden; 16 Wallenberg Center for Molecular and Translational Medicine, Lund University, Lund, Sweden; 17 Molecular Biophysics and Integrated Bioimaging, Lawrence Berkeley National Laboratory, Berkeley, CA USA; 18 Department of Psychiatry, School of Clinical Medicine, University of Cambridge, Cambridge, UK; 19 Wolfson Molecular Imaging Centre, Division of Neuroscience and Experimental Psychology, University of Manchester, Manchester, UK; 20 Queen Square Brain Bank for Neurological Disorders, UCL Queen Square Institute of Neurology, London, UK; 21 University of São Paulo Medical School, São Paulo, Brazil; 22 Global Brain Health Institute, San Francisco, CA USA; 23 Division of Clinical Geriatrics, Department of Neurobiology, Care Sciences and Society, Karolinska Institutet, Stockholm, Sweden; 24 Clinical Memory Research Unit, Department of Clinical Sciences, Faculty of Medicine, Lund University, Lund, Sweden; 25 King’s College London, Institute of Psychiatry, Psychology & Neuroscience, Maurice Wohl Clinical Neuroscience Institute, London, UK; 26 NIHR Biomedical Research Centre for Mental Health & Biomedical Research Unit for Dementia at South London & Maudsley NHS Foundation, London, UK; 27 Clinical Neurochemistry Laboratory, Sahlgrenska University Hospital, Mölndal, Sweden; 28 UK Dementia Research Institute at University College London, London, UK

**Keywords:** Biomarkers, Neurodegenerative diseases, Alzheimer’s disease, Tau, Amyloid, Neurofilament light chain, Magnetic resonance imaging, Positron emission tomography, Cerebrospinal fluid, Plasma biomarkers

## Abstract

**Background:**

The panel of fluid- and imaging-based biomarkers available for neurodegenerative disease research is growing and has the potential to close important gaps in research and the clinic. With this growth and increasing use, appropriate implementation and interpretation are paramount. Various biomarkers feature nuanced differences in strengths, limitations, and biases that must be considered when investigating disease etiology and clinical utility. For example, neuropathological investigations of Alzheimer’s disease pathogenesis can fall in disagreement with conclusions reached by biomarker-based investigations. Considering the varied strengths, limitations, and biases of different research methodologies and approaches may help harmonize disciplines within the neurodegenerative disease field.

**Purpose of review:**

Along with separate review articles covering fluid and imaging biomarkers in this issue of *Alzheimer’s Research and Therapy*, we present the result of a discussion from the 2019 Biomarkers in Neurodegenerative Diseases course at the University College London. Here, we discuss themes of biomarker use in neurodegenerative disease research, commenting on appropriate use, interpretation, and considerations for implementation across different neurodegenerative diseases. We also draw attention to areas where biomarker use can be combined with other disciplines to understand issues of pathophysiology and etiology underlying dementia. Lastly, we highlight novel modalities that have been proposed in the landscape of neurodegenerative disease research and care.

## Background

With 50 million individuals affected worldwide, neurodegenerative diseases remain without disease-modifying treatments. Neuropathological investigations are critical for closing intellectual gaps regarding pathophysiologic mechanisms, as postmortem examination serves as a gold standard and provides adequate resolution to observe the elements of pathophysiological cascades. However, they are limited to cross-sectional assessment. The use of antemortem biomarkers renders possible the detection of hallmarks longitudinally, throughout disease stages. Even in situations where conclusions drawn from antemortem biomarkers might differ from postmortem observations, antemortem biomarkers still offer utility. Here, we will illustrate ways that evidence can be weighed to understand neurodegenerative diseases holistically, focusing on methodology and the limitations and strengths of different modalities.

Despite sometimes indirect associations with lesions, neuropathologically validated biomarkers can be used to assess disease course, particularly as degeneration relates to clinical manifestations. For trials, biomarkers are of paramount importance. They are key for efficiently identifying and tracking cohorts by defining inclusion criteria and outcome variables [1]. As shown in a follow-up to an amyloid-β (Aβ) immunization trial [2], neuropathology can be informative for trials; however, the timelines associated with postmortem donation hinder drug development. As treatments emerge, biomarkers will become even more valuable as diagnostic tools.

There are numerous biomarkers for neurodegenerative diseases readily available or under development (Tables 1, 2, 3 and 4). In this issue of *Alzheimer’s Research and Therapy*, separate articles will discuss fluid (Obrocki et al.) and imaging (Young et al.) biomarkers for neurodegenerative diseases. Priming this, it is important to consider how one assesses the utility and biases of different modalities. We will discuss ways that biomarkers are utilized in neurodegenerative disease research and care, commenting on appropriate use, interpretation, and implementation. Finally, we will consider where major knowledge gaps lie and how novel biomarkers may fill them. This review will primarily focus on Alzheimer’s disease, due to a significant deficit in biomarkers useful for other neurodegenerative diseases; however, we will comment on emerging techniques for other diseases. Many of the themes discussed here on utility can be readily applied across neurodegenerative diseases.

**Table 1:** MRI biomarkers for neurodegenerative diseases

Biomarker	Target	Advantages	Disadvantages
Volumetry (vMRI)	Whole brain/medial temporal lobe, hippocampus	Highly reproducible and sensitive to disease-related changes	Late-stage biomarker; cannot provide information on the cause of atrophy
Cortical thickness	Cerebral cortex	May improve classification between dementia subtypes	Limited to neocortex; cannot be used to determine the cause of atrophy
Functional MRI (fMRI)	Regional/network functional activity	Can evaluate robusticity of networks at resting state and during tasks	Reproducibility and influence of vasculature unclear
FLAIR/T2 imaging	White matter lesions	Highly sensitive	Cannot determine the cause of the lesion
T2*/susceptibility-weighted imaging	Microbleeds/myelin/iron	Microbleed location can aid diagnosis	Undesirable artifacts at air/tissue interfaces; few longitudinal studies to date
Diffusion tensor imaging	White matter	Highly sensitive to white matter damage	Fairly low resolution and sensitive to artifacts from water diffusion; particularly sensitive to movements
Neuromelanin-sensitive	Locus coeruleus, substantia nigra	Sensitive to noradrenergic and dopaminergic subcortical nuclei	Semi-quantitative assessment/not disease specific

*Abbreviations*: *MRI* magnetic resonance imaging, *FLAIR* fluid-attenuated inversion recovery

**Table 2:** PET biomarkers for neurodegenerative diseases

Biomarker	Target	Advantages	Disadvantages
^18^F-FDG	Glucose metabolism	Capable of detecting dysfunction that has not necessarily involved atrophy; well-validated for clinical research	Variability in methods of analysis between studies/centers
^18^F-FDDNP	AD lesions	^18^F half-life does not require on-site cyclotron	Does not differentiate Aβ or tau aggregations
^11^C-PBB3	Tau lesions	Sensitive to both AD-type tau aggregations and non-AD tau aggregations; correlates with clinical progression	Short half-life of ^11^C; relatively low affinity compared to other tau markers; non-specific binding
^18^F-AV1451 (flortaucipir)	Tau lesions	High affinity to aggregated tau; distribution of binding reflects clinical presentations; ^18^F half-life does not require on-site cyclotron	Off-target binding to neuromelanin and choroid plexus; poor reliability in early NFT stages; preferential binding to mixed 3R/4R tau rather than isolated 3R or 4R tau
^18^F-GTP1	Tau lesions	Ligand binding maps to known distribution of AD-tau aggregations; ^18^F half-life does not require on-site cyclotron	Yet to be fully validated; possible high non-specific binding in basal ganglia
^18^F-MK6240	Tau lesions	Extremely high binding affinity to tau aggregations; good brain delivery and washout; ^18^F half-life does not require on-site cyclotron	Likely preferential binding to mixed 3R/4R tau rather than isolated 3R or 4R tau; off-target binding to neuromelanin
^18^F-RO6958948	Tau lesions	High binding affinity to tau aggregations; preliminary study shows longitudinal increases in AD; ^18^F half-life does not require on-site cyclotron	Yet to be fully validated; no significant binding in 3R or 4R tauopathies
^18^F-THK5351	Tau lesions	High binding affinity to tau over Aβ aggregations; ^18^F half-life does not require on-site cyclotron	High non-specific retention in the subcortical white matter; high MOA-B binding
^18^F-THK5105	Tau lesions	High binding affinity to tau over Aβ aggregations; ^18^F half-life does not require on-site cyclotron	High non-specific retention in the subcortical white matter; inferior signal-to-background ratio
^18^F-THK523	Tau lesions	High binding affinity to tau over Aβ aggregations; ^18^F half-life does not require on-site cyclotron	High non-specific retention in the subcortical white matter; poor in vivo visualization of tau deposition
^18^F-PI2620	Tau lesions	Binds to both 3R/4R mix tau and 3R tau (Pick’s disease); high binding affinity to tau over Aβ aggregations and MOA-A/MOA-B; ^18^F half-life does not require on-site cyclotron	Yet to be fully validated
^18^F-PM-PBB3	Tau lesions	Indication that ligand is sensitive to both AD and non-AD tauopathies; little binding to MOA-A and MOA-B; ^18^F half-life does not require on-site cyclotron	Yet to be fully validated, particularly in non-AD tauopathies
^11^C-PiB	Amyloid-β aggregations	High affinity to fibrillar Aβ; best studied of available Aβ PET tracers	Short half-life of ^11^C; not specific to AD amyloidosis/binds to CAA
^18^F-AV-45	Amyloid-β aggregations	High affinity to fibrillar Aβ; ^18^F half-life does not require on-site cyclotron	Not specific to AD amyloidosis
^18^F-BAY94-9172 (florbetaben)	Amyloid-β aggregations	High affinity to fibrillar Aβ; ^18^F half-life does not require on-site cyclotron	Not specific to AD amyloidosis
^18^F-GE067 (flutemetamol)	Amyloid-β aggregations	High affinity to fibrillar Aβ; ^18^F half-life does not require on-site cyclotron	Not specific to AD amyloidosis
^18^F-NAV4694	Amyloid-β aggregations	Excellent agreement with ^11^C-PiB; ^18^F half-life does not require on-site cyclotron	Not specific to AD amyloidosis
^11^C-UCB-J	Synapses (SV2A)	High affinity to protein expressed on synapses	Short half-life of ^11^C; relatively new tracer and not well-validated in dementia populations

*Abbreviations*: *PET* positron emission tomography, *FTD* frontotemporal dementia, *MOA-B* monoamine oxidase B, *PiB* Pittsburg Compound-B, *CAA* cerebral amyloid angiopathy, *FDG* fluorodeoxyglucose, *SV2A* synaptic vesicle glycoprotein 2A

**Table 3:** CSF biomarkers for neurodegenerative diseases

Biomarker	Target	Advantages	Disadvantages
Aβ42	Amyloid-β peptides	Strong correlation with ^11^C-PiB PET status	Different cutoff values used in different labs
Aβ40	Amyloid-β peptides	Added value when combined with Aβ42	Not a clinically meaningful biomarker in isolation
Aβ42/Aβ40 ratio	Amyloid-β peptides	Stronger diagnostic and prognostic value than Aβ42 alone	Not yet widely implemented
Aβ42/Aβ38 ratio	Amyloid-β peptides	Stronger diagnostic and prognostic value than Aβ42 alone	Not yet widely implemented
t-Tau	Tau peptides	Reasonably sensitive for late-stage AD	Poor specificity, not clinically useful in isolation
p-Thr181 tau	Tau peptides	Reasonably sensitive for late-stage AD	Poor specificity, not clinically useful in isolation
NfL	White matter damage	Indicates the presence of neurodegeneration	Increased in multiple neurodegenerative diseases

*Abbreviations*: *Aβ* amyloid-β, *NfL* neurofilament light chain

**Table 4:** Blood biomarkers for neurodegenerative diseases

Biomarker	Target	Advantages	Disadvantages
Aβ42/Aβ40 ratio	Amyloid-β peptides	Automated platform available for measurements	Difference in values between disease groups too small to be used as a diagnostic tool. Levels can be affected by pattern change from monomer to protofibrils in blood.
MDS-OAβ	Amyloid-β oligomers	Differentiates AD patients from HC with high sensitivity and specificity, not affected by pattern change in the blood	Poorly validated and limited availability of technology relative to ELISA-based methods
IIR assay	Amyloid α-helix versus β-sheet form	Detects biophysical properties of pathologic forms of amyloid instead of just concentrations	Poorly validated and limited availability of technology relative to ELISA-based methods
p-Thr181 tau	Tau peptides	Can accurately predict ^11^C-PiB PET status	Does not differentiate between tauopathies other than AD
t-Tau	Tau peptides	Automated platform available for measurements	Large overlap between diagnostic groups
NfL	White matter damage	Indicates the presence of neurodegeneration; strong correlation with CSF NfL	Increased in multiple neurodegenerative diseases

*Abbreviations*: *MDS-OAβ* Multimer Detection System-Oligomeric Amyloid β, *IIR assay* immune-infrared sensor assay, *NfL* neurofilament light chain

## Disease fundamentals

Neurodegenerative diseases represent the confluence of a range of pathophysiologic cascades with associated clinical spectra. While typically defined by proteinopathic hallmarks, it is unclear whether these hallmarks are driving the disease or are themselves consequences of other underlying processes. AD, frontotemporal lobar degeneration (FTLD), and Parkinson’s disease are the most common neurodegenerative diseases. Overlapping diseases, otherwise known as co-pathologies, are frequent and have complex contributions to clinical phenotypes [3].

### Alzheimer’s disease

Alzheimer’s disease (AD) is the most common cause of dementia with an incidence of 1700–2900/100,000 individuals per year in the USA [4]. AD is a dual proteinopathy characterized by the accumulation of tau neurofibrillary tangles and extracellular Aβ plaques [5]. Postmortem studies indicate that AD features a long preclinical phase where tau lesions and associated neuronal loss first appear in the subcortical nuclei and begin to involve limbic regions with associated subjective cognitive decline and neuropsychiatric symptoms [1, 6–9]. Cortical tau lesions appear in later stages [7], which correlate with the prototypical amnestic Alzheimer’s clinical syndrome [1, 10]. Also in the preclinical phase, Aβ lesions initially appear in the neocortical regions followed by the allocortical, then subcortical, and cerebellar involvement [11]. In contrast to tau lesions, the distribution of Aβ is not significantly associated with symptoms [12]. Besides age, risk factors include cerebrovascular diseases, diabetes, hypertension, obesity, dyslipidemia, and genetic factors such as the *APOE*-ε4 allele and *TREM2* mutations [13, 14]. Approximately 5% of cases have an age of onset under 65 and are referred to as early-onset AD cases. Familial AD, accounting for about 20% of early-onset cases and less than 1% of all AD cases, is mainly caused by rare, dominantly inherited *PSEN1*, *PSEN2*, or *APP* mutations (Table 5) [15–17].

**Table 5:** Genetic biomarkers for neurodegenerative diseases

Gene	Protein	Associated Syndrome(s)	Reference
*APP*	Amyloid precursor protein	EOAD (familial), CAA	Tanzi et al., 1987 [15]
*PSEN1*	Presenilin 1	EOAD (familial)	Sherrington et al., 1995 [16]
*PSEN2*	Presenilin 2	EOAD (familial)	Levy-Lahad et al., 1995 [17]
*MAPT*	MAPT	bvFTD, PSP	Hutton et al., 1998 [18]; Poorkaj et al., 1998 [19]
*C9orf72*	C9orf72	bvFTD, ALS	Renton et al., 2011 [20]
*GRN*	Progranulin	bvFTD, CBS	Gass et al., 2006 [21]
*VCP*	Valosin-containing protein	ALS	Johnson et al., 2010 [22]
*TARDBP*	TDP-43	ALS	Shreedharan et al., 2008 [23]
*SOD1*	Superoxide dismutase 1	ALS	Rosen et al., 1993 [24]
*FUS*	Fused-in sarcoma	ALS	Kwiatkowski et al., 2009 [25]; Vance et al., 2009 [26]
*HTT*	Huntingtin	HD	HDCRG, 1993 [27]
*SNCA*	α-synuclein	PD, DLB, MSA	Kruger et al., 1998 [28]
*GBA*	β-glucocerebrosidase	PD, DLB, Gaucher	Sidransky and Lopez, 2012 [29]
*PRNP*	Prion protein	Prion	Liao et al., 1986 [30]; Kretzschmar et al., 1986 [31]; Hsiao et al., 1989 [32]
*ApoE* (ε4 allele)	Apolioporotein-E	AD (risk factor)	Corder et al., 1993 [33]; Saunders et al., 1993 [34]
*TREM2*	TREM2	AD (risk factor)	Guerreiro et al., 2013 [35]; Jonsson and Stafansson, 2013 [36]

*Abbreviations: EOAD, Early-onset Alzheimer’s disease; MAPT, Microtubule associated protein tau; CAA, Cerebral amyloid angiopathy; bvFTD, Behavioral-variant frontotemporal dementia; PSP, progressive supranuclear palsy; ALS, Amyotrophic Lateral Sclerosis; TDP-43, Transactive response DNA binding protein 43 kDa; FUS, Fused-in Sarcoma; CBS, corticobasal syndrome; HD, Huntington’s disease; PD, Parkinson’s disease; DLB, Dementia with Lewy bodies; MSA, multiple system atrophy; AD, Alzheimer’s disease; TREM2, Triggering receptor expressed on myeloid cells 2*

### Parkinson’s disease

Synucleinopathies are the second most common neurodegenerative disease [18]. The most common clinical presentation of a synucleinopathy, Parkinson’s disease (PD), has an estimated incidence of 10–18/100,000 individuals per year in the USA [19]. Synucleinopathies feature the aggregation of α-synuclein in the form of Lewy bodies starting subcortically, spreading into limbic and neocortical regions [20]. Generally, synucleinopathies present as PD, PD dementia, or dementia with Lewy bodies, which collectively feature varying degrees of behavioral, cognitive, and autonomous symptoms on top of motor dysfunction. Mutations in a number of genes (Table 5) have been linked to synucleinopathies [10, 21], as has pesticide exposure and traumatic brain injury [22].

### Frontotemporal lobar degeneration

FTLD is nearly as prevalent as early-onset AD in dementia cases below the age of 65 with an incidence of 1.6–4.1/100,000 individuals annually in the USA and Europe [23, 24]. FTLD describes a spectrum of diseases characterized by vacuolation, gliosis, and neuronal loss in the external cortical layers of predominantly frontal and temporal neocortices and features the accumulation of tau, TDP-43, or fused-in sarcoma, which define the FTLD subtype [25, 26]. FTLD is associated with a spectrum of frontotemporal dementia (FTD) syndromes ranging from behavioral, language, to motor variants. Amyotrophic lateral sclerosis (ALS) and other motor neuron diseases can be considered part of the FTLD spectrum due to common neuropathologic and genetic features [27]. Roughly one third of all FTD cases are familial [28].

### Huntington’s disease

Huntington’s disease (HD) is a rare, autosomal dominant disease with an incidence of 0.16–0.94/100,000 individuals annually [29]. The age of onset of symptoms is typically in the mid-40s but can range from as young as 2 to over 80 and features chorea with behavioral and cognitive symptoms [30]. While there is a correlation between CAG repeat length and age of onset [31, 32], there is a high degree of variability making the predictive power of CAG repeat size poor, illustrating the complexity involved with treating HD. HD features progressive neuronal loss and astrogliosis in the striatum [33] as well as significant degeneration of the cortical regions, cerebellum, and brainstem nuclei [34]. While the huntingtin polyglutamate expansion leads to protein aggregation and transcriptional dysregulation, the mechanisms tying this to neuron loss are unclear.

### Prion diseases

Prion diseases are characterized by spongiform encephalopathy with underlying neuronal loss, gliosis, and aggregation of the prion protein (PrP) encoded by the *PRNP* gene [35]. PrP distinguishes prion diseases from other neurodegenerative diseases that may feature prion-like mechanisms, whereby misfolded amyloidogenic proteins spread throughout the brain via seeding aggregation processes. Several genetic, sporadic, and acquired/zoonotic forms of prion diseases exist with sporadic Creutzfeldt-Jakob disease (sCJD), accounting for roughly 85% of cases [36]. With a mortality rate of 1.5–2 individuals per million, sCJD is an extremely rare disease with an incidence rate that is difficult to ascertain [36]. Relevant clinical features include rapid cognitive decline, myoclonus, akinetic mutism, visual and cerebellar difficulties, and pyramidal or extrapyramidal features. A definitive identification requires neuropathological confirmation with postmortem or biopsied tissue. Probable identification requires either an electroencephalogram, magnetic resonance imaging (MRI), or cerebrospinal fluid (CSF)-based biomarkers with the use of real-time quaking-induced conversion (RT-QuIC) preferred over 14-3-3 detection [37].

## In vivo biomarkers for the study of pathophysiology and etiology

Decades of neuropathological studies augmented by novel biomedical tools have revealed that many complex, interrelated mechanisms underlie neurodegenerative diseases. A principle limitation of the cross-sectional nature of neuropathology is difficulty in assessing temporal relationships. Neuropathological investigations typically only observe end-stages; however, existing population-based cohorts can capture the full range of disease progression [38]. Detecting and addressing early phases of pathophysiological cascades are likely key for successful therapeutics; thus, a granular understanding of the early underlying biology and the identification of biomarkers representing these changes is paramount.

Given the clear etiology and sometimes predictable age of onset, several studies have focused on hereditary neurodegenerative diseases to investigate early disease stages. For example, positron emission tomography (PET) imaging studies of familial AD cases have suggested that brain Aβ deposition begins up to 20 years before clinical symptoms, while cortical tau deposition arises roughly 6 years before onset [39, 40]. Similarly, biomarker studies of FTLD mutation carriers suggest that pathological changes start decades before symptoms [41]. These studies demonstrate that a latency period exists prior to clinical decline which should be targeted therapeutically, confirming neuropathological observations. There is evidence that plasma Aβ42/40 is sensitive to preclinical amyloidosis, providing a more economical means than CSF or PET to identify participants in trials focused on early stages [42].

Curiously, conclusions from in vivo studies of the relative onset of Aβ and tau lesions in AD often conflict with investigations using large postmortem case series that indicate that tau lesions occur prior to Aβ deposition in the brain [6, 7]. This may be due to biases in the design of event-based modeling approaches with biomarkers. Young and colleagues [43] found that CSF levels of total tau and phosphorylated tau become abnormal prior to CSF levels of Aβ42, concurring with postmortem studies. It was not until they isolated the cohort to those who were already Aβ+, APOE+, or Aβ+ and APOE+, applying data-driven autopsy-validated cutoffs, which the sequence of biomarker changes recapitulated those typically found in biomarker studies. Further, differences between investigators regarding thresholds and characterization of what belongs to “clinically meaningful” disease spectra will affect the conclusions drawn. To illustrate, Weigand and colleagues found that Aβ PET-negative and tau PET-positive individuals tend to present with subtle cognitive changes, which they interpret to represent early AD-related changes [44]. That is to say that tau PET-positive and Aβ PET-negative individuals belong to an early phase of the disease spectra. This argument is strengthened by evidence that tau PET distribution predicts subsequent atrophy, whereas Aβ PET does not [45]. At the same time, others would characterize tau PET-positive and Aβ PET-negative individuals as having “suspected non-Alzheimer disease pathophysiology,” implying that they belong to a different disease spectrum instead of a, rather, early phase of AD that may later feature both lesions [1]. As the field remains split regarding the precise, biological definition of AD stages, interdisciplinary efforts must harmonize observations by acknowledging their varied, but complementary, strengths and limitations.

Information gleaned from longitudinal studies has been used to construct models of multiple biomarkers. The widely recognized “Jack curves” [46], for example, were explicitly intended to provide a “model of [AD] biomarkers” to help stage cases in vivo [46], setting a precedent for the 2018 guidelines for an in vivo research definition of AD [3]. However, the curves should not be interpreted as providing a comprehensive model of AD pathophysiology and are subject to revision as biomarkers become more advanced [46].

### Challenges in interpreting pathobiology

When combining biomarkers in a study, it is also important to account for differences in how they represent respective disease hallmarks. Importantly, the biomarkers must have a comparable target engagement with their respective hallmarks or, at the least, empirical measures of target engagement to adjust for different sensitivities. For fluid biomarkers, the kinetics of how biomarkers appear in the fluid samples (i.e., how a protein might be digested, processed, or passed into the CSF or blood) may differ and must be accounted for. While expensive and labor-intensive, kinetics can be measured through in vivo labeling techniques [47] that track protein production and clearance. Significant technological gaps exist with the detection of soluble biomarkers in vivo*.* Of note, lipid peroxidation and metabolism have been implicated in neurodegenerative diseases [48], yet current tools limit the degree to which this can be monitored in vivo*.* Similarly, robust fluid biomarkers of blood-brain barrier function have been lacking, with several under development.

Idiosyncratic properties of pathophysiological processes can manifest misleading results in biomarker-based studies of pathophysiology. For PET, there is a heterogeneous uptake of different ligands throughout the brain, and differences in this uptake, off-target binding, and imaging sensitivity should be accounted for. In AD, early tau lesions begin in the brainstem nuclei followed by limbic involvement, whereas Aβ lesions begin in the neocortical regions [7, 8, 49]. Several limitations with PET may lead to biases in detecting these two patterns. PET cameras are susceptible to partial volume effects and lack resolution to measure tau ligand binding in the subcortical regions, which are small but can have profound neurobiological and behavioral effects [6, 8, 9, 50–54]. Lowe and colleagues demonstrated that tau PET positivity denotes individuals who, at autopsy, are all already at Braak stage IV [55], at which point many subcortical structures and limbic regions would already feature significant degeneration [8, 9, 51]. Further, a tau PET imaging study [56], with relatively low SUVR values for positivity, estimated the prevalence of Braak stage 0 individuals as roughly 55% of their study population (*n* = 161) while a population-based autopsy study [6] featured just 28% of the study population (*n* = 455) as Braak stage 0. Depending on the thresholds used, tau PET recapitulates the histological progression of tau lesions seen at autopsy; however, the thresholds set may render the tau PET “Braak stage” an underestimate of the true Braak stage at autopsy, particularly at early stages, as seen in these studies [56, 57]. Additionally, some of the first regions to feature protein aggregation in neurodegenerative diseases contain neuromelanin, which many PET ligands will non-specifically bind to [58, 59]. By contrast, the signal from ^11^C-PiB and other ligands targeting Aβ can detect early, neocortical involvement of plaques [60], making Aβ easier to detect.

Other biological factors, including the significant impact of low-abundance hallmarks such as mutant huntingtin, synaptic proteins, and neurogranin should also be considered, as their low abundance may lead to underestimation of the roles these hallmarks play using event-based modeling approaches. To illustrate, differences in relative abundances of tau versus Aβ lesions, due to tau tangles accumulating within cells and Aβ occurring extracellularly, make the relative determination of proteinopathy onset difficult in vivo. The strengths of Aβ biomarkers may outweigh the weaknesses of tau biomarkers, potentially overrepresenting the relative involvement of Aβ versus tau lesions at different AD stages, especially in early stages. The development of biomarkers and studies with multidisciplinary, postmortem observations are needed to reconcile these issues.

Biomarkers are invaluable for elucidating pathophysiology with appropriate caution. Specific biological questions may call for different implementations of biomarkers, and establishing universal guidelines for their research use would be presumptuous. Biomarker status may be sensitive and specific to certain neuropathological diagnoses; however, one should not assume that clinical utility implies broad-sweeping research utility or vice versa. In vivo, labeling approaches and high-resolution biomarker-neuropathology correlations are critical for assessing the validity of individual biomarkers as they relate to underlying lesions [47]. Unless the extent of the factors connecting lesions to the detection of their corresponding biomarkers is elaborated, one must be cautious when employing biomarkers in studies of disease etiology, particularly when comparing biomarkers with varied biases.

## Biomarkers for the study of disease intervention

Rigorous validations of biomarkers through methods such as kinetics quantification [47] or postmortem correlation are necessary considerations for the selection of inclusion criteria and outcomes for clinical trials. Those trials that target underlying proteinopathy, such as the Aβ-modifying drugs for the treatment of AD, require an in vivo measurement of lesions to verify target engagement. By contrast, intervention studies that focus on specific symptoms of diseases, such as depression, may not require in vivo measurements of proteinopathy. Nevertheless, an operational, validated biomarker-based definition of different diseases is paramount for trials focused on disease-modifying therapies.

The 2018 NIA-AA guidelines for an in vivo research definition of AD set an outline for clinical trials based on a biomarker definition of AD [1]. The authors argue that since Aβ and tau lesions, together with neurodegeneration, define AD postmortem, participants should be identified in vivo based on these hallmarks. There remains disagreement regarding which biomarkers can be used to identify these hallmarks, though. Biomarkers for FTLD, synucleinopathies, and other neurodegenerative diseases are underdeveloped or have not yet been fully validated so a similar biomarker definition of other neurodegenerative diseases has yet to be established.

Aβ and tau PET imaging could serve as useful outcomes in clinical trials due to their ability to detect regional changes in vivo preceding significant atrophy [61, 62]. The implementation of tau PET in tau-targeting therapies is particularly encouraged due to the target’s associations with neurodegeneration and cognition [45, 63]. Further consideration regarding proteinopathy biomarker implementation in AD trials is warranted, though, as therapeutic changes in Aβ load have not associated with clinical changes or neurodegeneration [2]. Fluid levels of neurofilament light chain (NfL) are candidate outcomes for trials as they correlate with cognitive status and atrophy; however, they are not specific to underlying proteinopathy and instead represent the presence of general neurodegeneration [64].

Similarly, therapeutic development for HD serves to benefit from biomarker development. Despite the existence of a highly specific and sensitive genetic marker [62, 65], it is difficult to predict the age of onset [30]. Furthermore, clinical trials cannot rely on genetic markers as they will not change following a therapeutic intervention. Decreased striatal uptake of phosphodiesterase-10 PET tracer [66], atrophy in MRI [67], and plasma levels of IL-8, TNF-α [68], and NfL may be valuable markers for HD progression that could be used for evaluating the effectiveness of interventions.

Novel ultrasensitive immunoassays make blood biomarkers promising for the future as less invasive, cost-effective screening instruments [69]. In AD, plasma Aβ42 is decreased compared to controls [70, 71], and plasma Aβ42/40 is reduced in Aβ PET-positive individuals [72, 73]. Plasma NfL, which correlates with CSF NfL, may be useful for tracking neurodegeneration and associates with cognitive decline and atrophy [64, 74]. These require further validation but may serve as more accessible markers in the future [75]. Factors such as how plasma Aβ levels change in response to disease-modifying therapies, exposure time to drugs, and harmonization of analytical methodology remain a challenge [75, 76]. With well-characterized cohorts that now include Aβ PET and CSF measures, the opportunity to use an “endophenotype” approach to discover peripheral markers of lesions is increasing. Pilot data from discovery mass spectrometry and large panel-based approaches suggest associations of many plasma proteins and metabolites with AD [65, 77, 78]. However, these data should be interpreted with caution, as they are derived from multimarker panels with the mechanistic understanding of the associations lacking.

## Developing biomarkers for clinical use

In individuals with suspected neurodegenerative diseases that lack known monogenic etiologies, biomarkers are currently used on an exclusionary basis to rule out other diseases and may be used to support a specific diagnosis of a neurodegenerative disease. Other biomarkers are in the pipeline for clinical validation and determination of clinical value.

For example, structural MRI can be used to exclude space-occupying lesions and assess patterns of atrophy that aids diagnoses made by neurological assessment. Functional MRI and ^18^F-FDG PET can also be used to identify the affected brain regions and networks [10, 79, 80]. NfL is the best-established fluid marker for measuring neurodegeneration but has poor specificity [81]. A summary of currently available biomarkers that may hold clinical promise is provided in Tables 1, 2, 3 and 4.

Following the discovery and validation phases of biomarker development, standardization and clinical utility are key steps for clinical implementation. In these phases of biomarker development, key questions regarding optimal procedures, reproducibility, and elucidation of the biological relevance can be assessed. Further, these phases will determine the sensitivity and specificity of each biomarker to a given disease. For example, plasma NfL well represents the underlying neurodegeneration [74], but the consistency between labs is unclear and a great deal of overlap between diagnostic groups exists [81]. Another consideration in the standardization phase is the establishment of cutoffs for diagnostic thresholds. This requires a large study to assess biomarker values across diverse healthy and disease populations, accounting for possible differences in sample collection and analysis.

A further criterion for establishing biomarkers in clinical practice, even when biologically meaningful, is the utility for patient management which can determine financial coverage for the test. An ongoing study in the USA is examining how Aβ PET results influence patient management [82]. Preliminary results suggest that management is influenced by the use of Aβ PET; however, the effects on the prognosis are not yet known [82].

### New frontiers in biomarkers

In addition to the array of targets that can be detected by standard fluid and imaging biomarkers, other features of neurodegenerative diseases have been proposed as viable biomarkers of disease progression. One technique, the Multimer Detection System-Oligomeric Aβ, examines the tendency of plasma proteins to oligomerize, circumventing issues in measuring concentrations of Aβ itself [83]. Another uses biophysical properties associated with the propensity of the amyloid protein to form β-sheets to measure blood levels with an immune-infrared sensor assay [84, 85]. In vivo measurement of an early-affected region, the locus coeruleus, is being explored as a viable early biomarker using specialized MRI sequences and analysis [52]. In line with this, pupillometry is being explored as a measure of noradrenergic activity and, thus, locus coeruleus integrity [52]. Signal analysis from transcranial magnetic stimulation, electroencephalography, or magnetoencephalography has also shown promise as a biomarker of functional connectivity and may be sensitive to early changes [86–88]. Other functional readouts, such as sleep polysomnography and app-based digital phenotyping assessments have been proposed as early biomarkers as well [89–91].

## Conclusions

In combination with basic science and neuropathologic examination, biomarkers are invaluable for building a narrative on pathophysiology, clinical etiology, and strategies for interventions. As there are numerous ways biomarkers may be informative, caution must be exercised to ensure appropriate interpretation given the varied limitations of different modalities. Importantly, a significant gap remains for biomarker availability in non-AD neurodegenerative diseases. As the field works towards therapies for dementias, there continues to be a grave need for novel biomarkers, improved development of existing tools, standardization, and improved accessibility for clinical and research communities.

## Data Availability

Data sharing is not applicable to this article as no datasets were generated or analyzed during the current study.
