# Correction to: Relevance of biomarkers across different neurodegenerative diseases

**DOI:** 10.1186/s13195-020-00637-y

**Published:** 2020-06-09

**Authors:** Alexander J. Ehrenberg, Ayesha Khatun, Emma Coomans, Matthew J. Betts, Federica Capraro, Elisabeth H. Thijssen, Konstantin Senkevich, Tehmina Bharucha, Mehrsa Jafarpour, Peter N. E. Young, William Jagust, Stephen F. Carter, Tammaryn Lashley, Lea T. Grinberg, Joana B. Pereira, Niklas Mattsson-Carlgren, Nicholas J. Ashton, Jörg Hanrieder, Henrik Zetterberg, Michael Schöll, Ross W. Paterson

**Affiliations:** 1 Memory and Aging Center, Weill Institute for Neurosciences, University of California, San Francisco, San Francisco, CA USA; 2 Department of Integrative Biology, University of California, Berkeley, Berkeley, CA USA; 3 Helen Wills Neuroscience Institute, University of California, Berkeley, Berkeley, CA USA; 4 Dementia Research Centre, University College London Institute of Neurology, London, UK; 5 Department of Radiology & Nuclear Medicine, Amsterdam UMC, location VUmc, Amsterdam Neuroscience, Amsterdam, The Netherlands; 6 German Center for Neurodegenerative Diseases (DZNE), Magdeburg, Germany; 7 Institute of Cognitive Neurology and Dementia Research, Otto von Guericke University Magdeburg, Magdeburg, Germany; 8 The Francis Crick Institute, London, UK; 9 Department of Neuromuscular Diseases, University College London Queen Square Institute of Neurology, London, UK; 10 Department of Clinical Chemistry, Amsterdam UMC, Amsterdam, The Netherlands; 11 Petersburg Nuclear Physics Institute names by B.P. Konstantinov of National Research Center, Kurchatov Institute, St. Petersburg, Russia; 12 First Pavlov State Medical University of St. Petersburg, St. Petersburg, Russia; 13 Oxford Glycobiology Institute, Department of Biochemistry, University of Oxford, Oxford, UK; 14 Department of Neurodegenerative Disease, UCL Queen Square, Institute of Neurology, University College London, London, UK; 15 Department of Psychiatry and Neurochemistry, Sahlgrenska Academy at the University of Gothenburg, Mölndal, Sweden; 16 Wallenberg Center for Molecular and Translational Medicine, Lund University, Lund, Sweden; 17 Molecular Biophysics and Integrated Bioimaging, Lawrence Berkeley National Laboratory, Berkeley, CA USA; 18 Department of Psychiatry, School of Clinical Medicine, University of Cambridge, Cambridge, UK; 19 Wolfson Molecular Imaging Centre, Division of Neuroscience and Experimental Psychology, University of Manchester, Manchester, UK; 20 Queen Square Brain Bank for Neurological Disorders, UCL Queen Square Institute of Neurology, London, UK; 21 University of São Paulo Medical School, São Paulo, Brazil; 22 Global Brain Health Institute, San Francisco, CA USA; 23 Division of Clinical Geriatrics, Department of Neurobiology, Care Sciences and Society, Karolinska Institutet, Stockholm, Sweden; 24 Clinical Memory Research Unit, Department of Clinical Sciences, Faculty of Medicine, Lund University, Lund, Sweden; 25 King’s College London, Institute of Psychiatry, Psychology & Neuroscience, Maurice Wohl Clinical Neuroscience Institute, London, UK; 26 NIHR Biomedical Research Centre for Mental Health & Biomedical Research Unit for Dementia at South London & Maudsley NHS Foundation, London, UK; 27 Clinical Neurochemistry Laboratory, Sahlgrenska University Hospital, Mölndal, Sweden; 28 UK Dementia Research Institute at University College London, London, UK

**Correction to: Alz Res Ther 12, 56 (2020)**

**https://doi.org/10.1186/s13195-020-00601-w**

Following publication of the original article [1], the authors reported a production error in the article title. The last word "diseases" was omitted by copy editor.

The original article [1] has been updated.
